# Angiotensin II Signaling in Human Preadipose Cells: Participation of ERK_1,2_-Dependent Modulation of Akt

**DOI:** 10.1371/journal.pone.0075440

**Published:** 2013-10-02

**Authors:** Natalia Dünner, Carolina Quezada, F. Andrés Berndt, José Cánovas, Cecilia V. Rojas

**Affiliations:** 1 Institute of Biomedical Sciences, Faculty of Medicine, Universidad de Chile, Santiago, Chile; 2 Institute of Nutrition and Food Technology, Universidad de Chile, Santiago, Chile; INSERM, France

## Abstract

The renin-angiotensin system expressed in adipose tissue has been implicated in the modulation of adipocyte formation, glucose metabolism, triglyceride accumulation, lipolysis, and the onset of the adverse metabolic consequences of obesity. As we investigated angiotensin II signal transduction mechanisms in human preadipose cells, an interplay of extracellular-signal-regulated kinases 1 and 2 (ERK_1,2_) and Akt/PKB became evident. Angiotensin II caused attenuation of phosphorylated Akt (p-Akt), at serine 473; the p-Akt/Akt ratio decreased to 0.5±0.2-fold the control value without angiotensin II (p<0.001). Here we report that the reduction of phosphorylated Akt associates with ERK_1,2_ activities. In the absence of angiotensin II, inhibition of ERK_1,2_ activation with U0126 or PD98059 resulted in a 2.1±0.5 (p<0.001) and 1.4±0.2-fold (p<0.05) increase in the p-Akt/Akt ratio, respectively. In addition, partial knockdown of ERK_1_ protein expression by the short hairpin RNA technique also raised phosphorylated Akt in these cells (the p-Akt/Akt ratio was 1.5±0.1-fold the corresponding control; p<0.05). Furthermore, inhibition of ERK_1,2_ activation with U0126 prevented the reduction of p-Akt/Akt by angiotensin II. An analogous effect was found on the phosphorylation status of Akt downstream effectors, the forkhead box (Fox) proteins O1 and O4. Altogether, these results indicate that angiotensin II signaling in human preadipose cells involves an ERK_1,2_-dependent attenuation of Akt activity, whose impact on the biological functions under its regulation is not fully understood.

## Introduction

The renin-angiotensin system is known to play an important role in regulating cardiovascular and renal physiology. Recent evidence shows that renin-angiotensin systems also operate in diverse organs such as brain, pancreas, liver, gastrointestinal tract, and adipose tissue. Though, its specific functions in different tissues are not yet fully understood. Given that angiotensin II negatively influences systemic glucose metabolism and that augmented activity of the renin-angiotensin system is found in obesity, attention has lately focused on the effect of this hormone in adipose tissue.

Expression of the renin-angiotensin system components and the angiotensin II receptors in human adipose tissue was first described in subcutaneous fat [1]. Soon after, it was found that visceral fat presents the highest angiotensinogen expression, particularly in overweight subjects [2], [3], [4], [5], [6], [7]. In addition to renin and angiotensin converting enzyme (ACE), adipose tissue secretes other peptidases that can transform angiotensinogen into angiotensin II [8]. The enzymes that degrade the latter appear to participate in maintaining a tight control of local angiotensin II concentration [9]. Current investigations highlight the biological function of the new players, ACE2, angiotensin [1]–[7] and Mas receptor in the renin-angiotensin system [10].

The adipose tissue renin-angiotensin system appears to modulate triglyceride accumulation, lipolysis, inflammation, and adipogenesis [11]. A role for angiotensin II in the control of adipocyte formation first emerged from studies in transgenic mice [12]. Angiotensinogen deficient mice that were genetically modified to over express the gene encoding for the angiotensin II precursor polypeptide, solely in adipose tissue, exhibited a reduced number of adipocytes in their epididymal fat. Several investigations over the past years further supported a role for angiotensin II as a negative regulator of adipogenesis [1], [13], [14], [15], [16]. Angiotensin II inhibits the conversion of preadipose cells from subcutaneous [14], [15] and omental [13] adipose tissue into mature adipose cells. Of note, angiotensin II appears to exert a larger anti-adipogenic effect on preadipose cells from human obese subjects than on those from non-obese individuals [13]. Angiotensinogen expression is prominent in adipose cells from visceral fat from overweight individuals [2], [3], [4], [5], [6]. Interestingly, visceral fat preadipose cells (specially those from omental adipose tissue) are less prone to undergo adipogenic differentiation [17], [18]. It is conceivable that diminished adipocyte formation by angiotensin II may contribute to predominance of larger dysfunctional adipocytes in visceral fat, which associates with higher risk for cardiovascular disease and pathogenic metabolic alterations, such as impaired glucose tolerance, insulin resistance and chronic inflammation in human beings.

Angiotensin II signal transduction mechanisms have extensively been studied in cells from cardiovascular and adrenal systems, in which opposite physiological responses are triggered after binding type 1 (AT1) or type 2 (AT2) angiotensin II receptors. AT1 and AT2 receptors appear to participate in modulating adipocyte formation and function in mice and rats [19], [20]. Transcripts for both angiotensin II receptors have been detected in human visceral preadipose cells [21]. However, binding studies in preadipose cells and mature adipocytes from human adipose tissue only demonstrated presence of AT1 receptors [22], [23]. In addition, the AT1 receptor blocker irbesartan but not the AT2 receptor antagonist PD123319 overturned the inhibition of adipogenic differentiation by angiotensin II in preadipose cells from breast subcutaneous adipose tissue [14]. In agreement, our prior investigations showed that the AT1 receptor inhibitor losartan prevented angiotensin II reduction of adipogenesis in preadipose cells from human omental fat [13]. The canonical signaling mode of activated AT1 receptors involves heterotrimeric G proteins, namely, Gα_q11_ or Gα_i_ [24], [25]. However, angiotensin II signal transduction has proved to be far more complex than initially considered. Alternate AT1 receptor signaling pathways have also been described [25]. One of these signal transduction mechanisms involves interaction of AT1 receptors with β arrestin and G protein-coupled receptor kinases, which regulate the activity of mitogen-activated protein kinases (MAPK), specifically, ERK_1_ and ERK_2_. In addition, AT1 receptors are able to transactivate several members of the tyrosine kinase receptor family (such as PDGF, IGF-1 and EGF) in different cell types [26]. Phosphorylation of ERK_1_,_2_ by MAPK/ERK kinase 1 (MEK_1_) typically promotes translocation of these proteins into the nucleus where they regulate the activity of several transcription factors involved in growth and differentiation processes. Moreover, the presence of renin-angiotensin system components in intracellular compartments also associates with transcriptional regulation of gene expression [27], [28].

Participation of MAPK/ERK_1,2_ signaling in the response of human preadipose cell cultures to angiotensin II was previously reported [29]. We observed that exposure to angiotensin II increased phosphorylation of ERK_1,2_ and the key adipogenic transcription factor PPAR-gamma. An unanticipated attenuation of Akt phosphorylation was also detected, suggesting a connection between ERK_1,2_ and Akt activation. Integration of ERK_1,2_ and Akt signaling pathways has been found in numerous cell types in response to diverse stimuli, including angiotensin II [30], [31], [32], [33], [34], [35], [36], and can result in either positive or negative modulation of the pathway activity, depending on the biological context. Likewise, a cross-talk between ERK_1,2_ and Akt appears to operate in preadipose cells. Diverse inhibitors of adipogenesis, such as the polypeptide endothelin 1 [37] and the alkaloid evodiamine [38] have been reported to increase phosphorylated ERK_1,2_ and concurrently decrease phosphorylated Akt levels. Furthermore, chemical inhibition of ERK_1,2_ phosphorylation restores Akt activation; thus supporting the convergence of these signaling transduction pathways in preadipose cells.

Given that the phosphatidylinositol 3-kinase (PI3K)/Akt signaling pathway plays an important role in preadipocyte processes [39], [40], [41], [42], we sought to investigate the consequences of ERK_1,2_ activation by angiotensin II on Akt phosphorylation/activation in adipocyte precursor cell cultures obtained from human adipose tissue. This report shows that angiotensin II signaling involves an ERK_1,2_–dependent attenuation of Akt activity and reduced phosphorylation of Akt downstream effectors Fox O1 and Fox O4 in these cells.

## Materials and Methods

### Isolation and Culture of Preadipose Cells from Human Omental Adipose Tissue

Omental fat was obtained from 14 women that underwent elective abdominal surgery (gynecological procedures) at the Gynecology and Obstetrics Unit, Dr Luis Tisné Hospital. Institutional ethical committees at INTA, University of Chile, and Metropolitan Health Service, Santiago, Chile, approved the protocol. The donors signed informed consent. Women with malignancies or taking medications known to influence adipose tissue mass or metabolism were excluded. The mean body mass index of the donors was 34±5.7 Kg/m^2^. Routine blood analyses showed biochemical parameters within the normal range. Tolerance to glucose and sensitivity to insulin were not tested. Determination of basal phosphorylated Akt levels in this study provided no indication of altered sensitivity to insulin in cell cultures from any adipose tissue donor (the mean pAkt/Akt ratio in the whole group was 0.41±0.13; ranging from 0.36±0.10 to 0.43±0.11). Moreover, no association between the basal pAkt/Akt ratio and the donors’ BMI was found.

Fresh adipose tissue samples were immediately transported to the laboratory and the stromal-vascular cells were obtained as reported before [29]. Adherent cells were seeded in Dulbecco’s modified Eagle’s medium (DMEM)/Ham’s F12 (1∶1), containing penicillin, streptomycin (Invitrogen Corp, Carlsbad, CA), and supplemented with 10% fetal bovine serum (FBS, Hyclone, Thermo Scientific, South Logan, UT). Cells were grown on plastic culture dishes at 37°C in a humidified atmosphere with 5% CO_2_ until confluence was reached. Culture medium was replaced every 3 days. Expanded cultures were stored in liquid nitrogen for later use. Experiments were carried out after the second or third cell passage. Cell count was determined under a light microscope by means of a hemocytometer. To assess the phosphorylation status of proteins, cryopreserved stocks were seeded at 3.0 to 3.5×10^4^ cells/cm^2^. Given the variable yield of preadipose cells from different adipose tissue donors, the number of adipose cell samples included in each experiment is indicated in the figure legend.

### SGBS Cell Culture

The Simpson-Golabi-Behmel syndrome (SGBS) preadipose cells [43], generously provided by Dr Martin Wabitsch (University of Ulm, Germany), were used to assess the effect of angiotensin II on the phosphorylation status of FoxO1 and FoxO4 proteins. This clonal cell strain, obtained from a newborn with Simpson-Golabi-Behmel syndrome, has been validated as a model for human adipose cell studies [44]. These non-immortalized cells have the ability to proliferate for many generations, and to differentiate into cells with phenotypic and functional properties of mature adipocytes from healthy human subjects, including sensitivity to insulin. Their response to angiotensin II in adipogenic differentiation assays is similar to that of preadipose cell cultures from human adipose tissue (unpublished results). SGBS preadipocytes were expanded in DMEM/Ham’s F12 (1∶1) supplemented with 33×10^−3^ M biotin, 17×10^−3^ M sodium pantothenate, 0.1 mg/ml streptomycin, 100 U/ml penicillin, and 10% FBS.

### Determination of the Phosphorylation Status of ERK_1,2_, Akt, FoxO1 and FoxO4 Proteins

The relative level of phosphorylated ERK_1,2_ and Akt was evaluated to assess the signaling status of the corresponding intracellular pathways. To avoid a misleading effect of FBS components on the pathways’ activity, cultures were kept overnight with low (2%) FBS in DMEM/Ham’s F12 (1∶1), and maintained in FBS-free culture medium for 60 min before starting the experiments designed to test the effect of hormones or inhibitors. Given that phosphorylated Akt was barely detectable in this condition, the outcome of cell exposure to angiotensin II (alone or in combination with MEK inhibitors) was evaluated in FBS-free culture medium supplemented with human insulin. Hereafter, the basal culture medium contains 1.0×10^−6^ M insulin in DMEM/Ham’s F12 (1∶1).

When studying the effect of angiotensin II on the status of phosphorylated Akt and ERK_1,2_ under adipogenic conditions, adherent cells were also seeded at 3.0 to 3.5×10^4^ cells/cm^2^. Before stimulation of adipogenic differentiation, cells were incubated overnight with 2% FBS in DMEM/Ham’s F12 (1∶1). Adipogenesis was induced with 2.5×10^−7^ M dexamethasone (Sigma-Aldrich, St. Louis, MO, USA), and 5×10^−4^ M 3-isobutyl-1-methylxanthine (Sigma-Aldrich) in DMEM/Ham’s F12 (1∶1).

The dose-response to angiotensin II in human preadipose cells was previously determined [29]. Angiotensin was used at a supraphysiological concentration in order to compensate for the high rate of angiotensin II withdrawal detected in these cell cultures [29]. Angiotensin II concentration in all experiments was 1.5×10^−5^ M. Two cell-permeable MEK_1_ inhibitors, which prevent ERK_1,2_ activation through different mechanisms, were used to investigate participation of ERK_1,2_ activities in the response to angiotensin II. The compound 1,4-diamino-2,3-dicyano-1,4-bis (2-aminophenylthio) butadiene (U0126; Calbiochem EMD Biosciences Inc., San Diego, CA) directly inhibits MAPK/ERK kinase (MEK) activity [45]. Whereas the flavone derivative 2′-amino-3′-methoxy flavone (PD98059; Calbiochem EMD Biosciences Inc.) prevents MEK phosphorylation/activation [46], [47]. When indicated, culture medium was supplemented with 1×10^−5^ M U0126 or PD98059. The cell-permeable PI3K inhibitor 2-(4-morpholinyl)-8-phenyl-4H-1-benzopyran-4-one (Ly294002; Calbiochem EMD Biosciences Inc.) was used at 5×10^−5^ M. Inhibitors were added to the culture medium 60 min before angiotensin II, with dimethyl sulfoxide used as vehicle.

Cell lysates for Western blot analyses were prepared with 0.015 ml of RIPA buffer (consisting of 5×10^−2^ M Tris-HCl, 15×10^−2^ M NaCl, 1% Nonidet P-40, 0.5% sodium deoxycholate, 0.21% SDS, pH 8.0, supplemented with Complete™ protease inhibitors cocktail, Pepstatin A (Roche Applied Science, Mannheim, Germany) and Halt™ Phosphatase Inhibitor (Pierce, Thermo Scientific, Rockford, IL) per cm^2^ of culture dish area. Cell lysates were centrifuged at 4°C for 15 min at 5,000×g and supernatants were kept at −80°C until analyzed. Protein concentration was determined using the bicinchoninic acid method (Pierce, Thermo Fisher Scientific). Twenty to 60 micrograms of protein per sample were separated by 10% polyacrylamide gel electrophoresis under denaturing conditions and transferred to 0.45 µm Immobilon-P polyvinylidene difluoride membranes (Merck Millipore, Darmstadt, Germany). After protein transfer, nonspecific binding sites on the membranes were blocked with bovine serum albumin and probed with corresponding primary antibodies. Phosphorylated proteins were detected with antibodies (Cell Signaling Technology Inc, Danvers, MA) that recognize the specified phosphorylated amino acid residues in the corresponding target (ERK_1_ Thr202/Tyr204, ERK_2_ Thr185/Tyr187, Akt Ser473_,_ FoxO1 Ser256 and FoxO4 Ser262). Antibodies raised against a different epitope to determine abundance of total (phosphorylated and non-phosphorylated) matching proteins were also from Cell Signaling Technology Inc. The level of phosphorylated FoxO1 and FoxO4 proteins was expressed relative to β-tubulin because we were unable to immunodetect total FoxO proteins when blots were re-probed. The antibody specific for β-tubulin was purchased from Cell Signaling Technology Inc. The immune complexes were evidenced with appropriate horseradish peroxidase-conjugated secondary antibodies (Jackson Immunoresearch Laboratories, West Grove, PA) and enhanced chemiluminescence (Merck Millipore) was detected with the FOTO/Analyst Luminary/FX workstation (Fotodyne Inc., Hartland, WI). Phosphorylated to total protein ratios were calculated from digital images analyzed with the Total Lab TL100 software (Fotodyne Inc.) and expressed relative to the corresponding control in each experiment.

### Knockdown of ERK Expression by Posttranscriptional Gene Silencing

Lentiviral vectors carrying sequences for the expression of short hairpin RNAs (shRNA) designed to target ERK_1_ synthesis were generated. Based on mouse ERK_1_ short hairpin RNA [48], the following oligonucleotides were synthesized: 5′-GATCCCCGACCGGATGTTAA CCTTTATTCAAGAGATAAAGGTTAACATCCGTCTTTTTGGAAA-3′ and 5′-AGCTTTTCC AAAAAGACCGGATGTTAACCTTTATCTCTTGAATAAAGGTTAACATCCGGTCGGG-3′. The sequences in the human ERK_1_ gene (GenBank ID: NM002746) that create the stem of the shRNA are underlined. These oligonucleotides were annealed, and ligated into the lentiviral vector pFUGWH1 [49]. Viral stocks were prepared in 293T cells after cotransfection with the HIV-1 packaging vector pCMV-ΔR8.91 and the vector pVSV-G that expresses the envelope VSV-G glycoprotein [50], using the calcium phosphate method [51]. Viral particles in cell culture supernatants were harvested from 16 up to 84 h after transfection, concentrated by filtration through Ultracel-100 membranes (ULTRA-15™ filter units, Merck Millipore), and titrated by transducing HEK 293T cells with serial dilutions of the viral preparation. The percentage of GFP positive cells was determined 72 hours after infection by FACS analysis. The typical yield was 10^8^ viral particles per ml.

Transduction of primary preadipose cells was performed in 24-wells culture plates containing 8×10^4^ cells per well. Lentiviral particles (5.4×10^6^ per well) were added in 0.25 ml of DMEM/Ham’s F12 (1∶1) containing 10% SFB. Cells were incubated at 37°C in a humidified 5% CO_2_ atmosphere and the culture medium was replaced 16 h after viral infection. Transfection efficiency, monitored by green fluorescent protein expression, ranged from 70 to 80% in different experiments. To allow full expression of silencing shRNAs, cells were maintained in culture for 9 to 10 days, with renewal of the medium every three days. As indicated above, cells were kept overnight with 2% FBS in DMEM/Ham’s F12 (1∶1) and incubated in serum-free culture medium for 60 min before experiments were started.

### Data Analysis

All experiments were performed in duplicate and the number of samples from different adipose tissue donors included (n) is indicated in each case. Mean and standard deviation were determined. Pair wise comparisons were validated by Student’s t-test. To compare the response to parallel interventions one factor ANOVA was used, and followed by Tukey’s post hoc multiple comparison test. A probability (p) <0.05 was considered significant.

## Results

### Angiotensin II Reduces Phosphorylated Akt

**Figure 1:**
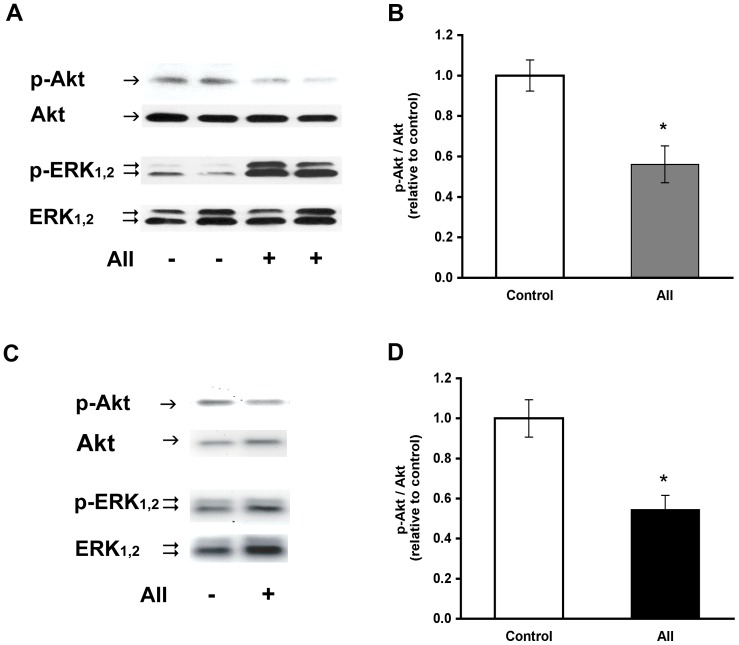
Reduction of phosphorylated Akt by angiotensin II. (A) Representative Western blot analysis of phosphorylated Akt (p-Akt), total Akt, phosphorylated ERK_1,2_ (p-ERK) and total ERK_1,2_ in extracts from cells incubated 8 h in adipogenic medium without (-) or with (+) 1.5×10^−5^ M angiotensin II (AII) during the last 2 h. (B) p-Akt/Akt ratios determined from Western blots in the conditions indicated in A and expressed relative to the value in the corresponding controls maintained in adipogenic medium without angiotensin II. The mean pAkt/Akt for the controls in these experiments was 1.56±0.27 (n = 5 donors). (C) The Western blots illustrate phosphorylated Akt (p-Akt), total Akt, phosphorylated ERK_1,2_ (p-ERK) and total ERK_1,2_ in extracts from cells maintained in basal culture conditions and treated without (−) or with (+) angiotensin II during 2 h. (D) The p-Akt/Akt ratios were determined from Western blots and normalized with respect to the corresponding controls in the absence of angiotensin II. The mean pAkt/Akt value for the control condition in these experiments was 0.43±0.11 (n = 8 donors). Blots in part A were overexposed to enhance low chemoluminescent signals for phosphoproteins at this time point. The symbol * denotes p<0.05.

We had previously demonstrated an increase in phosphorylated ERK_1,2_ when primary cultures of preadipose cells maintained in adipogenic differentiation medium were exposed to angiotensin II [29]. A concurrent decrease of phosphorylated Akt at serine 473 was initially noticed in this condition (Figure 1 A and B). Further analyses evidenced that the reduction of phosphorylated Akt after treatment with angiotensin II also occurred in the absence of adipogenic inducers, when cells were maintained in culture medium supplemented with fetal bovine serum or insulin. Hence, the following studies on the effect of angiotensin II on the phosphorylation status of ERK_1,2_ and Akt were carried out in the presence of insulin. Under this experimental condition, angiotensin II reduced the relative abundance of phosphorylated Akt, as illustrated in figure 1 (C and D). The p-Akt/Akt ratio was 0.5±0.2-fold that determined in the corresponding controls without angiotensin II (p<0.001, n = 8 donors).

### Inhibition of ERK_1,2_ Activation Increases Phosphorylated Akt

**Figure 2:**
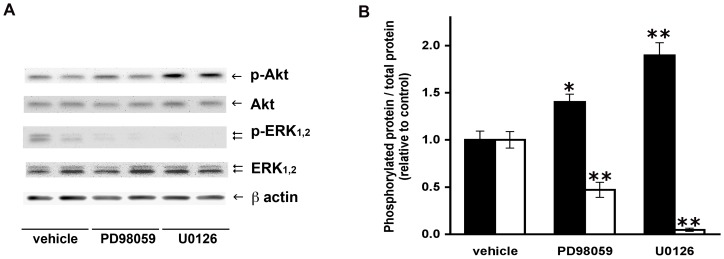
Increased phosphorylated Akt by inhibition of ERK_1,2_ activation. (A) Representative Western blots of phosphorylated Akt (p-Akt), total Akt, phosphorylated ERK_1,2_ (p-ERK), total ERK_1,2_ and β-actin in extracts from cells incubated for 60 min with 1×10^−5^ M U0126, 1×10^−5^ M PD98059 or vehicle. (B) The p-Akt/Akt ratios were determined from Western blots and values were normalized with respect to those determined in control cultures maintained in the basal medium supplemented with vehicle. The mean value for pAkt/Akt and p-ERK_1,2_/ERK_1,2_ in the controls were 0.41±0.08 and 1.53±0.30, respectively. The p-Akt/Akt (black bars) increase and the p-ERK_1,2_/ERK_1,2_ (white bars) decrease after incubation of cell cultures with the inhibitors of ERK_1,2_ activation (n = 5 donors). Symbols * and ** denote significant differences with respect to the control condition without inhibitors, at p<0.05 and p<0.01, respectively.

Chemical inhibition of ERK_1,2_ activation supported a connection with Akt phosphorylation in these cells. When ERK_1,2_ phosphorylation was prevented by treatment of cell cultures with U0126, a 2.1±0.5-fold rise in phosphorylated Akt was found (p<0.001; n = 9 donors), as illustrated in figure 2A. It is worth noting that Akt phosphorylation, which is undetectable in cells maintained without insulin, became apparent after exposure to U0126 in the absence of this hormone (data not shown). To rule out any nonspecific effect of U0126, the MEK inhibitor PD98059 was assessed in parallel. In consonance, both inhibitors of ERK_1,2_ activation reduced the fraction of phosphorylated ERK_1,2,_ as shown in figure 2A_._ The p-ERK_1,2_/ERK_1,2_ ratio was 0.47±0.18 and 0.05±0.04 relative to the control (vehicle) in cells that were incubated with 1×10^−5^ M PD980059 or 1×10^−5^ M U0126, respectively. As depicted in figure 2B, an increase in phosphorylated Akt was noticed in cells treated with both inhibitors. After exposure to 1×10^−5^ M PD980059 or 1×10^−5^ M U0126, the p-Akt/Akt ratio respectively was 1.4±0.2-fold (p<0.05) and 1.9±0.3-fold (p<0.01) the control value.

### ERK_1_ Knockdown Results in Increased Akt Phosphorylation

**Figure 3:**
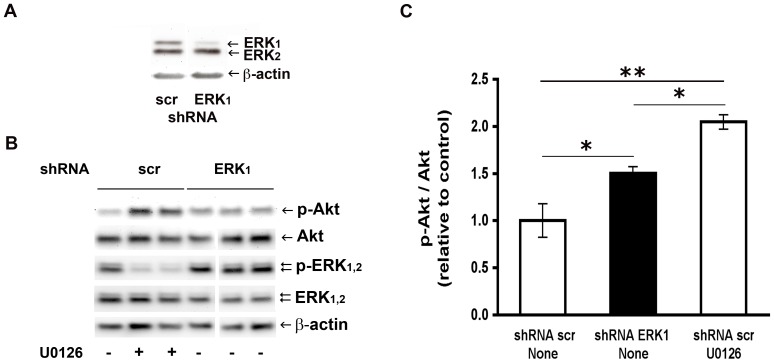
Increased phosphorylated Akt by ERK_1_ knockdown. (A) The Western blots illustrate ERK_1_, ERK_2_ and β-actin levels in cell lysates obtained 9 days after infection with lentiviral vectors carrying shRNA specific for ERK_1_ (shRNA ERK_1_) or a scrambled sequence (shRNA scr). (B) Representative Western blots of phosphorylated Akt (p-Akt), total Akt, phosphorylated ERK_1,2_ (p-ERK), total ERK_1,2_ and β-actin in cell cultures transduced either with ERK_1_ or scrambled shRNA constructs. When indicated (+), nine days after transduction with shRNA scr, cell cultures were exposed for 2 h to 1×10^−5^ M U0126 in basal medium. (C) The p-Akt/Akt ratios were determined from Western blots of cells transduced with shRNA scr (white bars) or shRNA ERK_1_ (black bar), and treated under the experimental conditions specified in part B, (n = 3 donors). The mean pAkt/Akt ratio in the control condition was 0.39±0.14. The symbols * and ** denote significantly different mean values, at p<0.05 and p<0.01, respectively (One-way ANOVA followed by Tukey’s post-hoc test).

To further uphold ERK_1,2_ involvement in the reduction of Akt phosphorylation, as suggested by the results with chemical inhibitors of ERK_1,2_ activation, we undertook the post transcriptional gene silencing approach with shRNA methodology. Given that only ERK_1_ appears to play an essential role in adipogenic differentiation [52], lentiviral vectors carrying the sequences for intracellular expression of shRNAs were designed to target ERK_1_ synthesis and thus to reduce the endogenous level of the ERK_1_ protein. Lower levels of ERK_1_ in primary cell cultures that were transduced with the lentivirus bearing the shRNA ERK_1_ construct were verified by Western blot analysis (Figure 3A). Nine days after infection, the ERK_1_/ERK_2_ ratio was 0.5±0.1 (range 0.4 to 0.7, p<0.0001, n = 5 donors) and the ERK_1_/β-actin ratio was 0.4±0.1-fold (range 0.2 to 0.6, p<0.0001, n = 5) with respect to control cells transduced with a scrambled sequence (no specific target). The reduced level of phosphorylated ERK_1_ in cells transduced with shRNA ERK_1_ was verified when expressed relative to β-actin. The p-ERK_1_/β-actin ratio was 0.5±0.1 (range 0.3 to 0.6, p = 0.004, n = 4 donors) versus the value in control cells transduced with a scrambled shRNA (shRNA scr), whereas the p-ERK_1_/ERK_1_ ratio was 1.4±0.2 the control value (range 1.2 to 1.6, p = 0.005, n = 4 donors).

The cells expressing shRNA ERK_1_ maintained under basal culture conditions showed an increased fraction of phosphorylated Akt, as illustrated in figure 3B. The p-Akt/Akt ratio was 1.5±0.1-fold the value determined in control cells transduced with scrambled shRNA (Figure 3C). This effect of ERK_1_ protein knockdown on the status of Akt phosphorylation was compared with that of chemical inhibition of ERK activation. To this end, the p-Akt/Akt ratio was determined in parallel in cells from the same donor, but transduced with shRNA scr and incubated in basal culture medium with or without the inhibitor U0126, as exemplified in figure 3B. The p-Akt/Akt ratio was 2.1±0.1 in cells with shRNA scr and treated with U0126 (Figure 3C). It is worth mentioning that exposure of the shRNA scr expressing cells to U0126 decreased the p-ERK/β-actin ratio to 0.3-fold the value in the absence of inhibitor; whereas the ratio was reduced to 0.5-fold in shRNA ERK_1_ expressing cells, as indicated above.

### Inhibition of ERK_1,2_ Activation Prevents Angiotensin II Attenuation of Akt, FoxO1 and FoxO4 Phosphorylation

**Figure 4:**
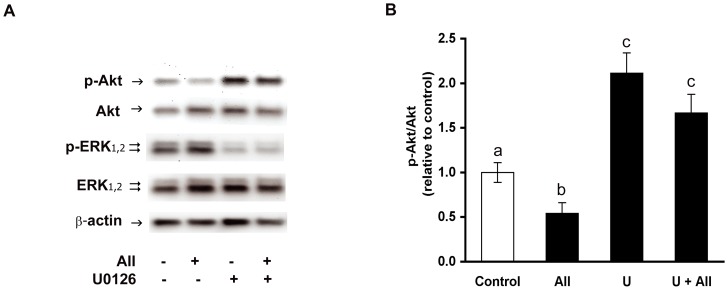
The inhibitor U0126 abolishes angiotensin II-dependent reduction of phosphorylated Akt. (A) Representative Western blot analysis of phosphorylated and total Akt in extracts from cells treated with 1×10^−5^ M U0126 for 60 min before exposure to angiotensin II (AII) for additional 60 min. Immunodetection of phosphorylated and total ERK_1,2_ illustrates p-ERK_1,2_ decrease after treatment with U0126. β-actin abundance was included as reference. The symbols+and – respectively denote presence or absence of AII or U0126 in the culture medium. (B) The p-Akt/Akt ratios were normalized with respect to the value measured in control cells without AII or U0126 (U). The mean pAkt/Akt value in the control condition was 0.36±0.10 (white bar). Different letters denote significantly different values, p<0.01; one-way ANOVA followed by Tukey’s post-hoc test; n = 4 donors).

**Figure 5:**
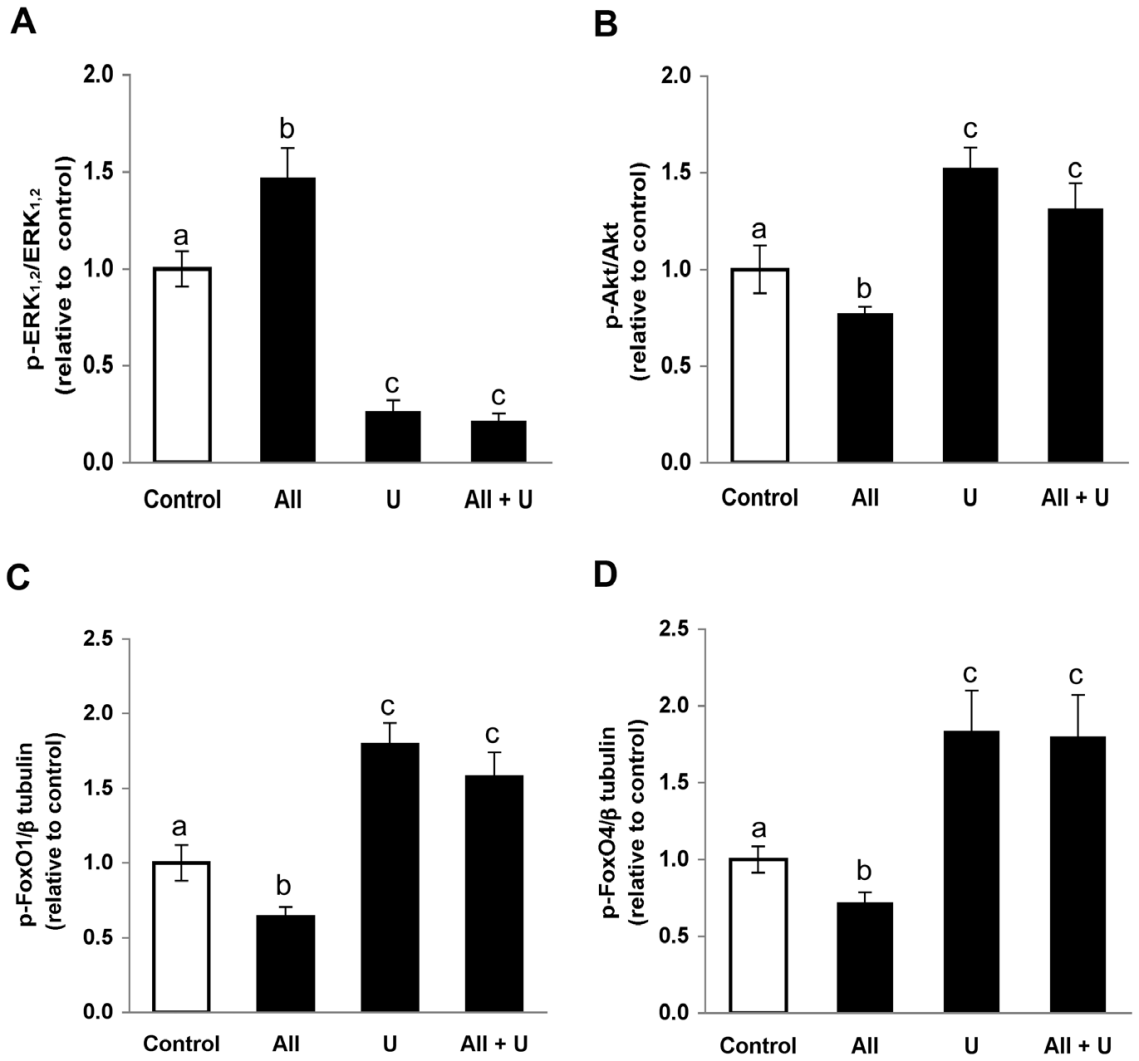
The inhibitor U0126 abolishes angiotensin II-dependent reduction of phosphorylated FoxO1 and FoxO4. SGBS cells were treated with 1×10^−5^ M U0126 (U) for 30 min before addition of angiotensin II (AII) and incubated in culture medium with insulin for additional 60 min. The phosphorylated level of ERK_1,2_ (A), Akt (B), FoxO1 (C) and FoxO4 (D) in cells treated with U plus AII were compared with those determined in cells treated solely with AII or U. The p-ERK_1,2_/ERK_1,2_ ratio and the p-Akt/Akt ratio in each experimental condition were expressed relative to the value measured in control cells without AII or U. Mean p-ERK_1,2_/ERK_1,2_, p-Akt/Akt values in the control condition were 0.87±0.17 and 0.40±0.12, respectively (white bars in A and B). Due to technical limitations, the p-FoxO1 and p-FoxO4 levels were expressed relative to β-tubulin abundance and normalized versus the corresponding value in the control condition specified above. Mean p-FoxO1/β-tubulin and p-FoxO4/β-tubulin ratios in the control condition were 0.14±0.04 and 0.37±0.07, respectively (white bars in C and D). Different letters denote significantly different values, p<0.05, n = 4 (One-way ANOVA followed by Tukey’s post-hoc test).

As mentioned above, the p-Akt/Akt ratio was reduced in primary preadipose cell cultures treated with angiotensin II. To test whether ERK_1,2_ activation is involved in attenuation of insulin-mediated phosphorylation of Akt by angiotensin II we made use of U0126, because the decrease of phosphorylated ERK achieved with this potent inhibitor was larger than that attained with the shRNA methodology. Pre-incubation with U0126 prevented a reduction of phosphorylated Akt by angiotensin II (Figure 4A). In those cells exposed to angiotensin II the p-Akt/Akt ratio was 0.5±0.2-fold the control value determined in the absence of this hormone. When angiotensin II was added to cells that had been pre-treated with 1×10^−5^ M U0126, the p-Akt/Akt ratio was 1.7±0.4 -fold that determined in the corresponding controls, which is comparable to the increased value (2.2±0.5-fold) found after treatment solely with U0126 (Figure 4B). Analogous results were observed when the phosphorylation status of downstream Akt effectors, FoxO1 and FoxO4, was evaluated after angiotensin II treatment under the experimental conditions indicated above. Because of limited availability of cells from adipose tissue donors, assessment of FoxO1 and FoxO4 phosphorylation was carried out with the SGBS cell strain, which show similar response to angiotensin II, with increased p-ERK_1,2_ (Figure 5A) and attenuated p-Akt levels (Figure 5B). Treatment of SGBS cells with angiotensin II resulted in decreased phosphorylation of FoxO1 (Figure 5C) and FoxO4 (Figure 5D), and this effect was averted by pre-treatment with U0126.

Taken together, these results indicate that reduced phosphorylation of Akt by angiotensin II depends on ERK_1,2_ activation. In contrast, inhibition of PI3K activity with LY294002, which decreased downstream Akt phosphorylation to 0.3±0.1-fold the corresponding control, had no effect on basal phosphorylated ERK_1,2_ levels. Moreover, the previously reported increase in the p-ERK_1,2_/ERK_1,2_ ratio by angiotensin II was not significantly modified by preincubation of the cells with 5×10^−5^ M LY294002 (data not shown); thus suggesting that PI3K activity is not involved in modulating ERK_1,2_ activity, under the experimental conditions here described.

## Discussion

Increasing evidence supports the complexity of angiotensin signal transduction. In this context, this study was designed to investigate a connection between ERK_1,2_ and Akt transduction pathways in the angiotensin II signaling mechanism, in preadipose cells from human omental fat. As shown above, inhibition of ERK_1,2_ activation by two structurally unrelated compounds (U0126 and PD98059) caused a 1.4 to 2-fold increase in the fraction of phosphorylated Akt protein (Figure 2), suggesting that ERK_1,2_ activities elicit a negative control over insulin-dependent Akt phosphorylation. It is noteworthy that phosphorylated Akt, which is barely detectable in cells maintained without insulin or SFB, became measurable after chemical inhibition of ERK_1,2_ activation under this condition (data not shown). The magnitude of the phosphorylated-Akt increase seems to relate to the extent of p-ERK_1,2_ reduction by U0126 or PD98059 (Figure 2). In addition, a diminished level of phosphorylated ERK, achieved by shRNA-mediated ERK_1_ knockdown, also caused a rise in the relative amount of phosphorylated Akt (Figure 3). Overall, these results endorse the hypothesis that the reduced level of activated ERK_1,2_ releases a negative control exerted on Akt phosphorylation/activation. This study did not elucidate the mechanism by which ERK_1,2_ down-regulates Akt kinase activity in these cells. Conceivably, activated ERK_1,2_ may control the activity of kinases or phosphatases that modulate Akt [32], [33], [36], [52]. Otherwise, ERK_1,2_ activities may determine the association of Akt with adaptor or scaffolding proteins, thus affecting its interaction with other regulators of intracellular signal transduction [53], [54].

As reported here, angiotensin II reduces insulin-dependent Akt phosphorylation at serine 473 and this effect is abolished by inhibition of ERK_1,2_ activation with U0126 (Figure 4). A coherent effect was revealed on typical downstream Akt effectors. Reduced phosphorylation of the transcription factors FoxO1 and FoxO4 was found after exposure to angiotensin II, and this effect was also prevented by treatment with U0126 (Figures 5C and D). The FoxO transcription factors, whose activity is regulated by protein-protein interactions and post-translational modifications (such as phosphorylation) have been implicated in diverse cellular processes, including glucose metabolism, adipocyte and muscle differentiation, oxidative stress response and inflammation [55]. Therefore, the outcome of reduced FoxO phosphorylation by angiotensin II in preadipose cells remains a matter of conjecture and deserve to be addressed in future investigations.

We had previously shown that angiotensin II treatment increased phosphorylated ERK_1,2_ [29]. Here we report that angiotensin II modulation of Akt phosphorylation in human preadipose cells appears to depend, at least in part, on ERK_1,2_ activities. In these cells, angiotensin II signaling resembles that of the antiadipogenic alkaloid evodiamine in the 3T3-L1 cell line [38]. In the latter evodiamine caused increased ERK phosphorylation and reduced insulin-stimulated phosphorylation of Akt, which were prevented by the MEK inhibitor PD98059. In addition, the authors report that upstream signals within the insulin/IGF-I pathway were not affected by evodiamine. Likewise, when bone marrow-derived mesenchymal stems cells were stimulated to adipogenic differentiation and exposed to high glucose, an ERK_1,2_-dependent increase in Akt phosphorylation was reported [56]. In these experimental conditions the PI3K inhibitor LY294002 did not affect the high glucose induced increase in ERK_1,2_ phosphorylation. Similarly, we found no evidence of PI3K activation upstream of angiotensin II-dependent ERK_1,2_ phosphorylation in human preadipose cells (data not shown). In contrast, MAPK-ERK and PI3K-Akt pathway interactions appear to involve PI3K-dependent ERK_1,2_ phosphorylation in other cell types [32], [35], [36], [57], [58]. We did not investigate other intracellular mechanisms reportedly involved in angiotensin II signal transduction in different cell types. Therefore, we cannot rule out their participation in the inhibition of Akt activation reported here.

In addition to the acknowledged functions of angiotensin II as a regulator of vascular and renal homeostasis, attention has been lately paid to its putative role in the onset of resistance to insulin. Clinical studies have shown that angiotensin-converting enzyme inhibitors as well as angiotensin II receptor blockers improve whole body insulin sensitivity [59]. In accordance, angiotensin II appears to modulate insulin sensitivity in diverse cell types, such as endothelial [31], hepatic [32] smooth and skeletal muscle [60], [61], [62]. Moreover, in a model of fructose-induced insulin-resistant rats, administration of the AT1 receptor antagonist candesartan cilexetil led to improvement of the defects in dorsal root ganglia neurons associated with impaired AT2 receptor function [63]. On this ground, it is tempting to speculate that increased angiotensinogen secretion by omental adipose tissue from obese individuals [3], [4], [5], [6], [7] would result in a high local angiotensin II concentration and likely, in reduced Akt phosphorylation in adipose and preadipose cells. In agreement with this idea, diminished Akt phosphorylation at serine 473 has been found in adipocytes from subjects with insulin resistance [64]. Moreover, reduced phosphorylation at serine 473 has also been reported in preadipose cells from human omental fat of obese but non-diabetic subjects [65]. Therefore, reduced phosphorylation at Akt serine 473 may predispose to impaired sensitivity to insulin in adipose tissue. Given the complexity of Akt interactions with many intracellular pathways that regulate a broad range of cellular processes it is unlikely that the outcome of impaired Akt phosphorylation by angiotensin II would be restricted to insensitivity to insulin.

In conclusion, we report an ERK_1,2_ -dependent attenuation of Akt phosphorylation/activity in the response of human preadipose cells to angiotensin II. Given the involvement of angiotensin II in modulation of diverse processes, such as oxidative stress, inflammation, insulin sensitivity, and cellular differentiation, a comprehensive understanding of the underlying pathways in specific target cells should provide the bases for the design of new therapeutic strategies.
